# Early reappearance of intraclonal proliferative subpopulations in ibrutinib-resistant chronic lymphocytic leukemia

**DOI:** 10.1038/s41375-024-02301-y

**Published:** 2024-06-24

**Authors:** Federico Pozzo, Gabriela Forestieri, Filippo Vit, Giulia Ianna, Erika Tissino, Tamara Bittolo, Robel Papotti, Annalisa Gaglio, Lodovico Terzi di Bergamo, Agostino Steffan, Jerry Polesel, Pietro Bulian, Roberta Laureana, Agostino Tafuri, Annalisa Chiarenza, Francesco Di Raimondo, Jacopo Olivieri, Francesco Zaja, Luca Laurenti, Maria Ilaria Del Principe, Massimiliano Postorino, Giovanni Del Poeta, Riccardo Bomben, Antonella Zucchetto, Davide Rossi, Valter Gattei

**Affiliations:** 1 Clinical and Experimental Onco-Hematology Unit, Centro di Riferimento Oncologico di Aviano (CRO), IRCCS, Aviano, 33081 Italy; 2 Experimental Hematology, Institute of Oncology Research, Bellinzona, 6500 Switzerland; 3 Immunopathology and Cancer Biomarkers, Centro di Riferimento Oncologico di Aviano (CRO), IRCCS, Aviano, 33081 Italy; 4 Unit of Cancer Epidemiology, Centro di Riferimento Oncologico di Aviano (CRO) IRCCS, Aviano, 33081 Italy; 5 Department of Biomedicine and Prevention, Hematology, University Tor Vergata, Rome, 00133 Italy; 6 Hematology Unit, Azienda Ospedaliera-Universitaria Sant’Andrea, Rome, 00189 Italy; 7 Division of Haematology, Ferrarotto Hospital, Catania, 95124 Italy; 8 Hematology Clinic, Azienda Sanitaria Universitaria Friuli Centrale (ASUFC), Udine, 33100 Italy; 9 Department of Medical, Surgical and Health Sciences, University of Trieste, Trieste, 34127 Italy; 10 Institute of Hematology, Fondazione Policlinico Universitario Agostino Gemelli IRCCS, Rome, 00168 Italy

**Keywords:** Translational research, Chronic lymphocytic leukaemia

## Abstract

The Bruton’s tyrosine kinase (BTK) inhibitor ibrutinib represents an effective strategy for treatment of chronic lymphocytic leukemia (CLL), nevertheless about 30% of patients eventually undergo disease progression. Here we investigated by flow cytometry the long-term modulation of the CLL CXCR4^dim^/CD5^bright^ proliferative fraction (PF), its correlation with therapeutic outcome and emergence of ibrutinib resistance. By longitudinal tracking, the PF, initially suppressed by ibrutinib, reappeared upon early disease progression, without association with lymphocyte count or serum beta-2-microglobulin. Somatic mutations of *BTK*/*PLCG2*, detected in 57% of progressing cases, were significantly enriched in PF with a 3-fold greater allele frequency than the non-PF fraction, suggesting a *BTK/PLCG2*-mutated reservoir resident within the proliferative compartments. PF increase was also present in *BTK/PLCG2*-unmutated cases at progression, indicating that PF evaluation could represent a marker of CLL progression under ibrutinib. Furthermore, we evidence different transcriptomic profiles of PF at progression in cases with or without *BTK/PLCG2* mutations, suggestive of a reactivation of B-cell receptor signaling or the emergence of bypass signaling through MYC and/or Toll-Like-Receptor-9. Clinically, longitudinal monitoring of the CXCR4^dim^/CD5^bright^ PF by flow cytometry may provide a simple tool helping to intercept CLL progression under ibrutinib therapy.

## Introduction

In the last years, the Bruton’s tyrosine kinase inhibitors (BTKi) including ibrutinib [1, 2] widely proved to be effective for both treatment-naïve and relapsed/refractory chronic lymphocytic leukemia (CLL). Nevertheless, it is well known that BTKi-based therapies are rarely curative and most of the patients eventually discontinue treatment due to either toxicity or disease progression, the latter associated with the emergence of resistance mutations on the *BTK* and *PLCG2* genes [3, 4]. Biologically, BTK inhibition interferes, within the tumor microenvironment, with different pathways required for CLL cell survival and proliferation such as the B-cell receptor (BCR) [5, 6], integrins [7] and cytokines/chemokines receptors [8, 9]. Such an impairment, resulting in the loss of the supportive nodal environment, causes a “redistribution lymphocytosis”. i.e., the rapid relocation of leukemic cells from the lymphoid tissues to the blood stream where they undergo senescence and eventually apoptosis [9–12].

In the peripheral blood, the reciprocal expression of CD5 and CXCR4 (CD184) characterizes different subpopulations that derive from the turnover/recirculation of CLL cells between the lymph nodes and the blood stream: a proliferative fraction (CXCR4^dim^/CD5^bright^, PF), recently divided and egressed from the lymph node, and a resting fraction (CXCR4^bright^/CD5^dim^, RF) of older, quiescent cells [13] .The current model supports the idea of a linear advancement from the PF to the RF, which then re-enters the lymph node niche upon CXCL12/SDF-1 gradient, to undergo further cycles of proliferation [14–17].

Despite the known efficacy of BTKi to inhibit CLL proliferation in the short term [5, 6], little is known about the impact of long-term BTKi treatment on the PF/RF dynamics. Herein, we demonstrated that upon clinical progression on ibrutinib, the PF is reconstituted enriched in *BTK/PLCG2* mutations, suggesting the presence of active clonal selection dynamics taking place within the lymph node microenvironment.

## Materials and methods

### Study cohorts and primary CLL cells

The study is part of a comprehensive CLL characterization approved by the Internal Review Board of the Centro di Riferimento Oncologico di Aviano (Approvals n. IRB-05-2010, n. IRB-05-2015 and n. CRO-2017-31) upon informed consent in accordance with the declaration of Helsinki and included CLL patients from two cohorts: (1) 31 patients enrolled in the IOSI-EMA-001 trial (NCT02827617) of ibrutinib monotherapy, with longitudinal collection of peripheral blood (PB) samples (IOSI cohort); (2) 101 CLL patients treated with ibrutinib in the current clinical practice, referred to the Clinical and Experimental Onco-Hematology Unit of the Aviano National Cancer Institute for molecular and cytogenetic analyses (CRO cohort).

The IOSI cohort included 156 PB samples, a median of 6 samples/case (range 3–7), collected at specified time-points: pre-treatment and 0.5–6–12–18–24 months after ibrutinib initiation. Seven cases discontinued ibrutinib due to progression (*n* = 4), infection (*n* = 2) or intolerance (*n* = 1). The CRO cohort included 300 PB samples, a median of 3 samples/case (range 2–7), up to 8 years of ibrutinib treatment (Fig. S1A). Seventy-nine out of 101 cases discontinued ibrutinib, with a median time-to-discontinuation (TTD) of 39.8 months (range 1–96; Fig. S1B), due to toxicity (*n* = 13), progression (as per iwCLL criteria, ref. [18], *n* = 53), other reason (e.g., secondary neoplasia, infection, clinical decision; *n* = 13; Fig. S1C). TTD per discontinuation category was 16.1 months for toxicity (range 1.0–32), 40.8 months for progression (3.0–82.5), 42.8 months for other reason (e.g., secondary neoplasia) or death (1.3–99.5). Sixty-four out of 79 patients had a “near-discontinuation” PB sample, i.e., collected within 12 months before/after ibrutinib discontinuation (Fig. S1D). Of these cases, 10 discontinued due to toxicity, 47 because of progression and 7 for other reasons or death. The remaining 22 patients were still under treatment at the time of analysis, with a median time under ibrutinib of 41.5 months (range 23–96) and a median time between last sample and last follow-up of 15.6 months (range 0–79.2). Samples were categorized according to the time from ibrutinib initiation to sampling; in presence of multiple close samplings (e.g., weekly/monthly evaluation of lymphocytosis upon ibrutinib initiation), only one representative sample was kept. Pre-ibrutinib samples were defined as collected within 6 months before treatment initiation. Samples collected before ibrutinib initiation (Fig. S1A) were included to provide a baseline for the PF evaluation in an ibrutinib-free setting.

PBMC were isolated from PB samples by Ficoll-Hypaque (GE Healthcare, Uppsala, Sweden) density gradient centrifugation and either used directly or cryopreserved until use. CLL cases were characterized for IGHV mutational status, the main cytogenetic abnormalities and mutational status of recurrently mutated genes as described [19, 20]. Patients’ characteristics are reported in Table S1.

### Immunophenotype

For the CRO cohort, immunophenotype was performed on fresh whole blood at sample’s arrival, as part of the routine diagnostic evaluation; for the IOSI cohort, it was performed on cryopreserved cells immediately after thawing. Samples were stained with a multicolor panel (CD19, CD5, CXCR4, CD49d, DAPI) and homogenously acquired on a 5-laser LSR Fortessa (IOSI cohort), or 3-laser FACSCantoII analyzer (CRO cohort) upon daily instrument calibration with CS&T beads. Acquisition was set for about 10 000 CLL cells and, for low cellularity samples, at least 1000 CLL cells. Patient-wise gating of the PF/RF subpopulations was performed with respect to the 80/90^th^ percentiles of event density distribution of CD5 and CXCR4, in comparison with the pre-therapy samples and accounting for inter-patient variability, and reviewed by two independent investigators. Data were analyzed with FacsDiva (v.6-9) or FlowJo (v.10.9) software. All reagents, instruments and software were from BD Biosciences (Franklin Lakes, NJ).

### Cell sorting

Cryopreserved CLL cells from selected cases were thawed and immediately stained with the following antibodies: CD19-BB700, CD49d-PE, CD5-FITC, CXCR4-APC, DAPI, CD3-APC-H7 and sorted according to CD5/CXCR4 expression with a FACSAriaIII cell sorter (BD Biosciences). Gates were manually set for each sample around the CD5/CXCR4 dim and bright populations. Nucleic acids were immediately extracted from sorted cells using either the AllPrep DNA/RNA extraction kit or the RNeasy Micro kit (Qiagen, Hilden, Germany) according to manufacturer’s instructions, and quantified with Nanodrop (ThermoFisher Scientific, Waltham, MA).

### Next Generation Sequencing (NGS)

Targeted DNA sequencing on sorted PF/RF subpopulations was performed on *TP53* (exons 2 to 11) [20], *NOTCH1* (exon 34 and 3’UTR) [21], *SF3B1* (exons 12-13-14-16) and *BIRC3* (exons 6-7-9) [22, 23], *BTK* (exons 11-15-16) and *PLCG2* (exons 12-19-20–24-27-30) [24], as previously reported [20]. Briefly, at least 40 nanograms of DNA were amplified with gene-specific PCR primers (designed and modified according to Illumina, San Diego, CA) and a Phusion High-Fidelity DNA Polymerase (ThermoFisher Scientific), purified with Purelink PCR purification kit (ThermoFisher Scientific), quantified with Quantifuor ONE dsDNA (Promega, Madison, WI), and paired-end sequenced in a MiSeq v2 600 cycles flow cell (Illumina). Data were analyzed with MiSeq Reporter (Illumina) against human genome assembly hg19. Variants with “Pass” flag were annotated with Annovar [25] and visualized with IGV software [26]. Results were expressed as Variant Allele Frequency (VAF).

### Copy number variation analysis

*MYC* amplification was investigated using droplet digital PCR (Bio-Rad, Hercules, CA) with FAM-labeled *MYC* and VIC-labeled *TERT* TaqMan Assay probes (Hs02758348_cn and 4403316; ThermoFisher Scientific) [27]. Fluorescence In-Situ Hybridization was performed with a *MYC* break-apart probe (MetaSystems, Heidelberg, Germany) according to manufacturer’s protocol. Controls included a normal reference DNA (Female reference, Promega), a sample of Richter Transformation with confirmed *MYC* amplification, and the *MYC*-hyperamplified HL-60 cell line.

### In-vitro functional assays

BCR stimulation was performed as reported [7] on thawed cryopreserved cells. Stimulations included: bead-conjugated anti-IgM (SureBeads, Bio-Rad; goat anti-human IgM, Southern Biotech, Birmingham, AL) in a 1:1 cell:bead ratio; 0.1 µg/mL CD40 ligand (MegaCD40L, Enzo life Sciences, Farmingdale, NY); 7.5 µg/mL CpG-oligodinucleotide (Integrated DNA Technologies, Coralville, IA) with 100 U/mL IL-2 (R&D Systems, Minneapolis, MN). Phosphoflow was performed with anti-pBTK and anti-pERK antibodies as reported [7].

### RNA sequencing (RNA-seq)

RNA-seq was carried out on sorted PF/RF from 12 patients, 8 of which with matching samples at pre-ibrutinib and progression, while in 4 patients only samples taken at progression were available. Libraries were prepared with the mRNA Library prep kit (Illumina) according to manufacturer’s protocol from at least 20 ng of total RNA. Briefly, 20–50 ng of total RNAs from sorted cells was poly-A purified, fragmented, and first-strand cDNA reverse transcribed using random primers, following second-strand cDNA synthesis, adapter ligation, and PCR amplification. Integrity and library size were assessed with Agilent Tapestation (Agilent Technologies, Santa Clara, CA). Libraries were pooled and sequenced on a NovaSeq S4 v1.5 100 cycles flow cell (Illumina). RNA-seq reads were mapped on human assembly build hg38 with STAR v.2.7.3a [28] using default parameters and *--quant* mode with gencode_v34 annotation for gene counts. RNA-sequencing data can be accessed to Gene Expression Omnibus at record GSE249956.

### Data mining tools

Bioinformatic analyses were performed using R v.4.3.0 (www.r-project.org) and R/Bioconductor software packages in RStudio. Differentially expressed genes were identified using the *DESeq2* package v.1.40.1 [29], upon prefiltering with the *FilterByExpression* function from *edgeR* package v.3.42.4, using Benjamini-Hochberg correction for multiple testing, and an adjusted *p* < 0.01 and a fold change >2 unless otherwise specified. Patient IDs were included as an independent variable to remove batch effects to due inter-patient variability. Comparisons for fold change direction were defined as PF versus RF, or progression versus pre-ibrutinib. Heatmaps of log2 normalized expression values were visualized as row-Z-scores using the *ComplexHeatmap* package v.2.16.0 [30], with unsupervised hierarchical clustering with euclidean distance and complete linkage. Gene Set Enrichment Analysis (GSEA, http://www.broad.mit.edu/gsea/index.jsp) [31] was performed with GSEA-4.2 java platform using 1000 gene set permutations with default parameters. Gene Set Variation Analysis (GSVA) was performed with the *GSVA* Bioconductor Package v.3.17 [32]. For both analyses, expression dataset of all detected genes within each comparison was used as input, without prior pre-ranking. Investigated gene sets were either collected from the Molecular Signature Database v6.2 (http://software.broadinstitute.org/gsea/msigdb) [33] or manually curated from indicated publications. Gene sets were assessed as significantly enriched in one of the phenotypes if the nominal *p* value and the FDR-q value were less than 0.05.

### Statistical analyses

Statistical analyses were performed using Medcalc software and R. Data were compared using two-sided Mann–Whitney rank-test and presented with Tukey’s box-and-whiskers plot. A *p*-value smaller than 0.05 was considered as significant and represented with asterisks: *p ≤ 0.05, **p ≤ 0.01, ***p ≤ 0.001, ****p ≤ 0.0001; non-significant comparisons were not reported. For clinical evaluation, time-to-discontinuation (TTD) was defined as the time between BTKi treatment initiation and discontinuation for any reason (event) or last follow-up (censoring). Probability of treatment discontinuation was estimated by the Kaplan–Meier method, with log-rank test used to compare probabilities between subgroups. To account for the competing risk of discontinuation, risk of progression was evaluated through cumulative incidence; ibrutinib discontinuation for toxicity or other causes were computed as competing events, and differences across PF thresholds were tested through Gray’s test [34]. Analysis was carried out with the *tidycmprsk* package v.0.2.0.

## Results

### Ibrutinib efficiently suppresses the PF until disease progression

**Fig. 1:**
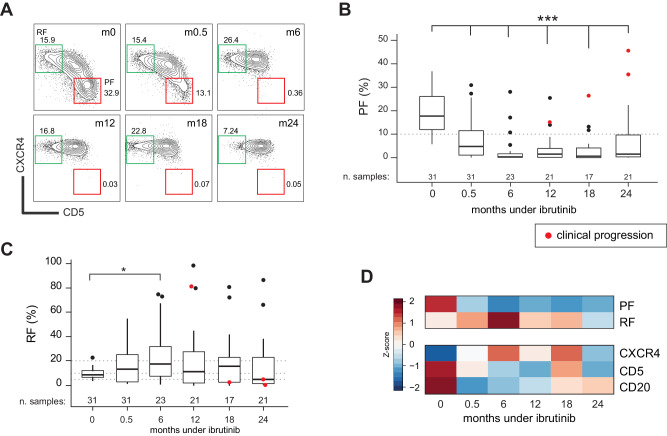
Proliferative fraction dynamics in the IOSI-EMA-001 trial. **A** Gating strategy for proliferative (PF, CXCR4^dim^/CD5^bright^, red) and resting (RF, CXCR4^bright^/CD5^dim^, green) populations by flow cytometry of a representative CLL case in the IOSI cohort. Populations were tracked using a fixed-gate strategy. Reported are percentage of each population over the entire CD19 + /CD5 + CLL population and the month of sampling under ibrutinib. Trend of the proliferative fraction (PF, **B**) and resting fraction (RF, **C**) over time by month under ibrutinib. ***p ≤ 0.001 by two-sided Mann–Whitney rank-test compared to month zero. Only significant comparisons are reported. Red dots indicate samples collected from patients close to clinical progression. **D** Heatmap summarizing the row-normalized median value of investigated features over the cohort: percentage of parent population for PF/RF, mean fluorescence intensity for CXCR4, CD5, and CD20.

The dynamics of the PF were monitored by flow cytometry in the IOSI cohort through a patient-specific, fixed-gate strategy (Fig. 1A and Fig. S2) [13]. The PF and RF were quantified as a percentage of the CD5 + /CD19 + CLL cell population. As reported for early time points under ibrutinib [8], the PF showed a rapid depletion, from a median of 17.0% at pre-treatment (range 6.5–37.0) to 4.3% at two weeks and to 0.4% at six months, remaining stable at 1.7%, 0.6%, 1.5% at subsequent time points (weeks 48, 72, 96; range 0–50.2; *p* < 0.001 for all comparisons with pre-treatment; Fig. 1B and Fig. S3A); concordantly, the loss of the CXCR4^dim^ population reflected in an increase in CXCR4 mean fluorescence intensity (Fig. S3B). The RF presented an inverse trend, with an increase at 6 months (8.6% vs. 18.2%, *p* = 0.024; Fig. 1C and Fig. S3C), and with a noticeable, although not significant, decrease in CD5 expression (Fig. S3D). Conversely, CD20 expression showed a more marked modulation and was effectively reduced by ibrutinib (Fig. S3E), in agreement with previous studies [8, 35]. Four cases had a clinical progression within the timeframe of the study, showing the highest values of PF (Fig. 1B, red marks). A summary of the over-time modulation of PF, RF, CD5, CXCR4, and CD20 expression is reported in Fig. 1D.

### Reappearance of PF associates with CLL progression under ibrutinib

**Fig. 2:**
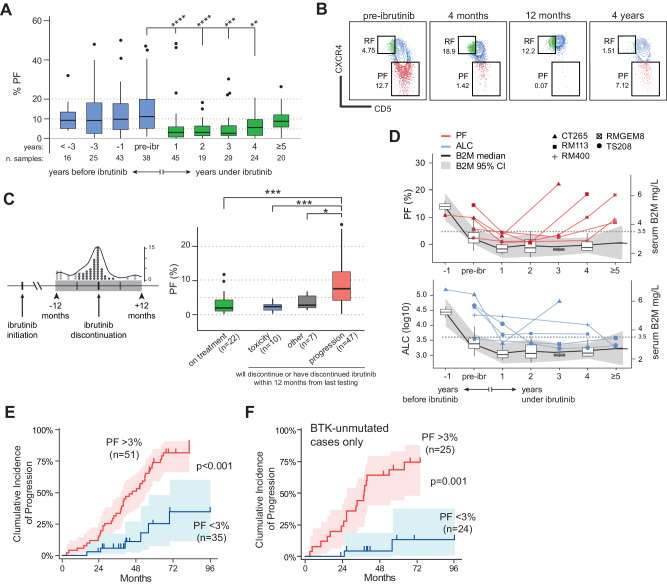
Proliferative fraction dynamics under long-term ibrutinib in the CRO cohort. **A** Box-and-whiskers plot of the PF in samples from the CRO cohort before (blue) and after (green) ibrutinib initiation. Samples are grouped by number of years under ibrutinib; pre-ibrutinib samples have been collected within 6 months prior to therapy initiation. Dotted lines are added for visual reference. ****p ≤ 0.0001, ***p ≤ 0.001, **p ≤ 0.01 by two-sided Mann–Whitney rank-test compared to pre-ibrutinib. Only significant comparisons are reported. **B** Representative flow cytometry contour/dot plot of patient RM113 showing reppearance of the PF after four years of ibrutinib therapy. Contours denote 10% steps of event density. **C** Left panel: binned dot and density distribution of 64 samples collected within 12 months from ibrutinib discontinuation (“near-discontinuation samples”); right panel: distribution of the PF of the near-discontinuation samples, split by cause of discontinuation, compared to the 22 on-treatment samples (overall *n* = 86). Dotted lines are added for visual reference. ***p ≤ 0.001, by two-sided Mann–Whitney rank-test. Only significant comparisons are reported. **D** Trend for beta-2-microglobulin (B2M) serum levels (boxplots and gray area) and PF (red lines) or absolute lymphocyte count (ALC, blue lines) in five patients progressed under ibrutinib. Dotted line represents the 3.5 mg/L threshold for B2M. **E** Cumulative incidence of progression, split by PF above (red) or below (blue) the ROC criterion in the near-discontinuation cohort (*n* = 86; PF > 3%). Reported is *p* value of Gray’s test. **F** Curve of cumulative incidence of progression split by PF above (red) or below (blue) the ROC criterion (PF > 3%) in *BTK*-unmutated cases only (*n* = 49). Reported is *p* value of Gray’s test.

To extend our observations to longer periods, we analyzed the PF dynamics in the CRO cohort of 101 ibrutinib-treated CLL cases from the real world. Median PF of samples at pre-ibrutinib was 11.0% (range 0.9–47.0), as estimated in 122 samples from 92 patients within 3 years before ibrutinib start (Fig. 2A). Upon ibrutinib treatment, the PF dropped to 2.9% after one/two years and to 2.0% after three years (range at 1 y 0–55%; range at 2 y 0–18%; range at 3 y 0–30%; *p* < 0.001 for all comparisons; Fig. 2A, B); examples of the gating strategy are reported in Fig. S4A. Absolute lymphocyte count (ALC) count followed a similar trend, decreasing from an average of 30 000 lymphocytes to about 5000 lymphocytes /µL at three years (Fig. S4B). Stratification by IGHV status in both PF and ALC, confirmed the equal capacity of ibrutinib to inhibit cell proliferation (Fig. S4C) with no significant differences between IGHV-mutated and unmutated cases.

At later time points of ibrutinib therapy (4 and 5 years), a rebound of the PF became prominent, with a median increase to 4.9% at 4 years (range 0.1–20.0; *p* = 0.003) and to 8.2% at five or more years (range 0.2–26.1; *p* = 0.303; Fig. 2A, B). On the contrary, the RF did not show any significant evolution over time (Fig. S4D), in agreement with a more relevant role for CXCR4, one of the main mediators of the CLL recirculation [17, 36–38], over CD5.

To verify whether this rebound was associated with clinical progression, we focused on the 64 “near-discontinuation” samples, i.e., PB samples collected within 12 months before/after ibrutinib discontinuation (Fig. 2C, left panel), and compared with the last sample from 22 patients still on treatment. Stratification by cause of discontinuation revealed a significantly higher PF in clinically progressed cases compared to cases who discontinued ibrutinib because of toxicity (median 8.4% vs. 2.2%, *p* < 0.001) or to those still on treatment (median 1.8%, *p* < 0.001; Fig. 2C right panel and Fig. S5A).

ALC and serum beta-2-microglobulin (B2M) levels are key laboratory markers of disease burden [39–42]. The PF in the near-discontinuation samples did not show any correlation with ALC levels, even if stratified by cause of discontinuation (Fig. S5B). We then quantified the modulation of B2M levels in progressed cases with enough samples to infer a time trajectory (*n* = 5); whilst B2M levels remained steadily low during ibrutinib, below the 3.5 mg/L threshold (Fig. 2D), the PF showed an evident emergence at earlier time points (Fig. 2D upper panel). A similar trend was present also for the ALC values which, with only one exception, maintained steady levels while under ibrutinib (Fig. 2D, lower panel). Receiver Operating Characteristic (ROC) analysis of the PF versus probability of progression selected a criterion of PF > 3% as the most discriminating for a higher risk of progression, with a sensitivity of 86.7% and a specificity of 71.1% (Fig. S5C); application of this 3% cut-off for the PF was able to significantly separate patients into subgroups with a higher incidence of early progression (74 vs. 25% at 5 years, *p* < 0.001; Fig. 2E). Of note, mean fluorescence intensity of CXCR4 or CD20, as a surrogate marker of ibrutinib efficacy, although respectively up-regulated and down-regulated, were not able to discriminate progressing cases from those still on therapy (Fig. S5D)

### Elevated PF at progression associates with *BTK/PLCG2* mutations

We hypothesized that the PF re-emerging at disease progression, as recently egressed from the nodal compartments, could be enriched in mutations driving ibrutinib resistance [3, 4]. Mutations of *BTK/PLCG2* were detected in 31/101 cases; of these, 29 (93%) discontinued ibrutinib due to progression and 2 cases because of other reason or death. Median time of appearance of *BTK/PLCG2* mutations was 48 months (range 16–70). Median variant allele frequency (VAF) was 22.8% for *BTK* (range 1.2–99.0) and 3.5% for *PLCG2* (range 1.7–17.6).

**Fig. 3:**
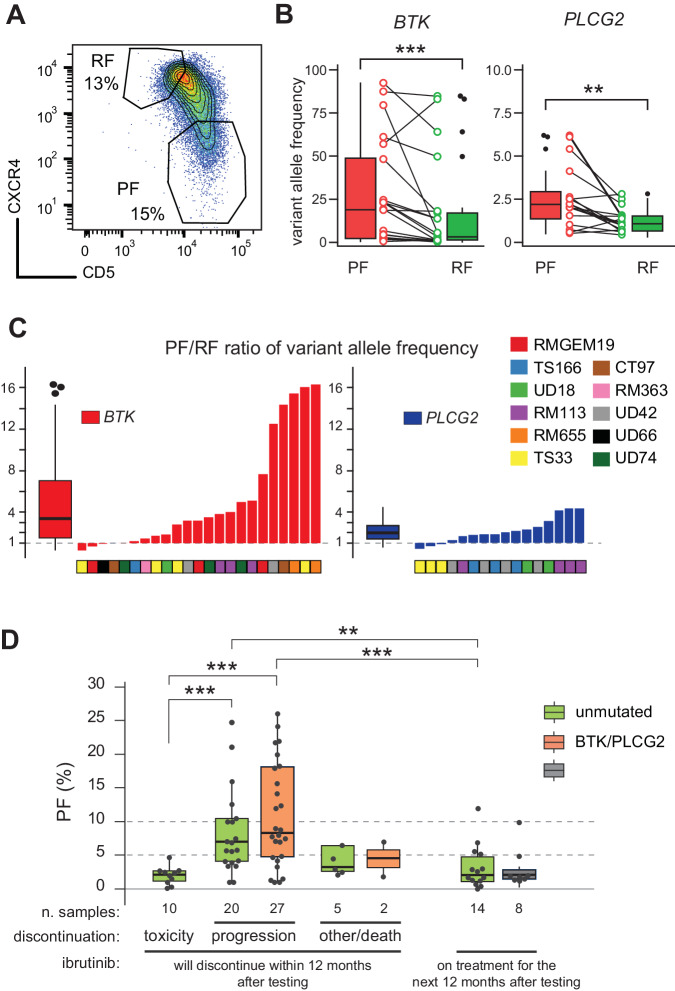
Elevated PF associated with *BTK* mutations. **A** Flow cytometry dot plot of cell sorting strategy for PF/RF fractions. **B** Box-and-whiskers and dot-and-line plot for variant allele frequency (VAF) of *BTK/PLCG2* mutations in 11 CLL samples collected near clinical progression. ***p ≤ 0.001, **p ≤ 0.01, by paired Wilcoxon rank test. **C** Bar plot of PF over RF VAF ratios of each *BTK* (red) or *PLCG2* (blue) mutation (*n* = 39 for 11 patients) found in all sequenced fractions. Box plots summarize the overall distribution of the ratios. **D** Box-and-whiskers plot of the PF in the near-discontinuation samples (*n* = 86), split by presence of *BTK/PLCG2* mutations. ***p ≤ 0.001, **p ≤ 0.01 by two-sided Mann–Whitney rank-test. Only significant comparisons are reported.

By fluorescence-activated cell sorting, PF and RF were isolated (Fig. 3A) from 11 progressed cases bearing *BTK/PLCG2* mutations, who presented a reappearance of the PF after prolonged treatment (median TTD 50.7 months). Of note, PF cells were significantly larger than RF cells (143 vs. 109 forward scatter intensity, *p* < 0.0001) and yielded a higher RNA content (0.23 vs. 0.08 picograms of RNA per cell, *p* = 0.0002; Fig. S6A), in keeping with an ongoing proliferation [43]. DNA targeted sequencing revealed a total of 22 *BTK* mutations (average of 2 mutations per case, range 1–4), with a median VAF in the PF of 18.7% (range 0.3–92.3), higher than in the RF (median VAF 2.15%, range 0.1–84.8, *p* = 0.0002 Wilcoxon paired rank test; Fig. 3B). Overall, the *BTK* VAF in the PF was about 3 times larger than the RF (median ratio 3.36, range 0.28–16; Fig. 3C). A total of 17 accompanying *PLCG2* mutations were present in 6 out of 11 cases, generally more frequent (average 2.8 mutations per case, range 1–4) but with lower VAF, although higher in PF than in RF (median in PF: 2.2%, range 0.5–6.2; median in RF: 1.1%, range 0.4–2.8; *p* = 0.002 Wilcoxon paired rank test; Fig. 3B, C). Finally, we could not find an enrichment of other recurrent genetic lesions, including *TP53*, *NOTCH1*, *BIRC3,* and *SF3B1* [20–24], in the PF or RF (Fig. S6B, C).

When we stratified the 64 near-discontinuation samples (Fig. 2C) by *BTK/PLCG2* status, mutated progressing cases (27/47, 57%) presented a PF significantly higher than cases discontinuing for toxicity (8.4% vs. 2.2%; *p* < 0.001) or still on treatment (2.1%, *p* < 0.001).

Notably, however, also the 20 (63%) unmutated progressing cases showed PF higher than cases discontinuing for toxicity (6.4% vs. 2.2%; *p* < 0.001) or cases still on treatment (6.4% vs. 2.1%; *p* < 0.001; Fig. 3D), but not different from mutated cases (*p* = 0.193). In fact, also in these *BTK/PLCG2*-wild type cases, the PF evaluation, according to the 3% criterion, identified a subset of patients at higher incidence of progression (Fig. 2F). Overall, these data indicate that the newly reconstituted PF at progression is associated with *BTK/PLCG2* mutations, suggesting that the reservoir of resistant cells is likely nested within the lymph node niches, to be later released in the blood stream. Our data further confirm that a significant fraction of progressing cases cannot be identified solely by presence of resistance mutations, whereas evaluation of the PF may represent a potential marker of CLL progression under therapy with ibrutinib.

### Transcriptomic programming of the PF is maintained at disease progression

To verify whether the expression profile of the PF and RF subpopulations from 12 cases. Fractions were cell-sorted from eight cases with matching samples at pre-ibrutinib and progression, and four cases with samples at progression only, all IGHV-unmutated, and analyzed by mRNA sequencing; notably, the immunophenotype immediately upon thawing was highly consistent with the one acquired on the fresh sample(Fig. S7A, B).

**Fig. 4:**
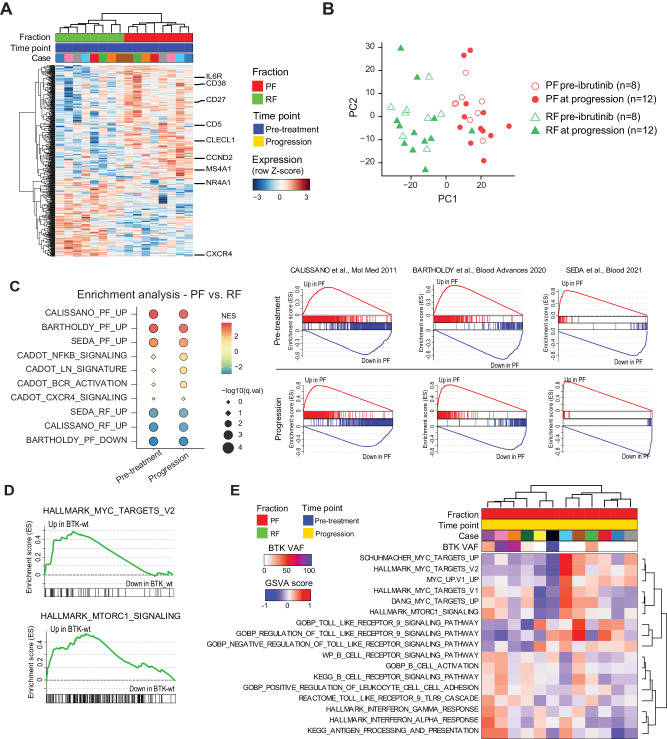
Transcriptomic programming of the PF/RF subpopulations. **A** Clustered heatmap of 476 differentially expressed genes between paired PF and RF in 16 fractions from 8 CLL patients before ibrutinib initiation. Gene list is reported in Table S2. **B** Principal component analysis (PCA) of RNA-seq of all sorted fractions (*n* = 40) using the 476 genes signature, splitting PF (red) from RF (green) samples. **C** Summary dot-plot of Gene Set Enrichment Analysis (GSEA) on PF/RF-related gene sets curated from literature (Calissano et al. ref. [13], Bartholdy et al. ref. [15], Cadot et al. ref. [46], Seda et al. ref. [17]). Color represents the normalized enrichment score, size is proportional to –log10(FDR q-value), shape denotes significance (circle if *q* < 0.05). Enrichment plots of selected gene sets are reported separately for samples at pre-ibrutinib and at progression. GSEA summary is reported in Table S3A. **D** Enrichment plot from GSEA of the HALLMARK_MYC_TARGETS_V2 and HALLMARC_MTORC1_SIGNALING of *BTK*-unmutated (*BTK*-wt) versus *BTK*-mutated PF at progression. GSEA summary for whole Hallmark collection is reported in Table S4C. **E** Heatmap of Gene Set Variation Analysis (GSVA) of *BTK*-unmutated (*BTK*-wt) versus *BTK*-mutated PF at progression, run with selected gene sets.

Differential expression (DE) signature of PF vs. RF at pre-treatment identified 479 genes, 285 up-regulated in the PF, and 194-downregulated in the PF (Fig. 4A, Table S2). RNA-seq counts of *CD5* and *CXCR4* transcripts were able to recapitulate the same PF/RF phenotypic distribution as flow cytometry, suggesting a direct correlation between transcript and protein expression for these molecules (Fig. S7C). Among other DE genes, we could highlight the differential modulation of *CCND2*, *TJAP, GAB1,* and *CCR7* [15, 17], the up-regulation in PF of *CD27* and *CLECL1*, two genes recently associated with the CLL proliferative phenotype [44, 45] as well as of *MS4A1/*CD20 [8] (Fig. S7C).

This signature, when applied to samples collected at progression, could again clearly separate PF/RF by Principal Component Analysis (PCA; Fig. 4B) and clustering (Fig. S7D), with 19 out of 20 PF/RF isolated at progression co-segregating with their respective pre-treatment counterparts (Fig. S7E). We further investigated this similarity running GSEA, using different published proliferation-related signatures [13, 15, 17, 46]. These proliferation-related gene sets were strongly enriched in pre-treatment samples but remained also significantly enriched at progression, suggesting that these transcriptional programs were comparably active (Fig. 4C and Table S3A). These data confirm that the PF at progression is a true novel reconstituted PF, functionally similar to the original pre-treatment PF.

We then investigated the impact of ibrutinib treatment on the transcriptome, running GSEA with several published signatures of DE genes characterizing ibrutinib sensitivity [10, 46–49]. Evaluation of these signatures in the combined fractions, comparing progression versus pre-ibrutinib, showed very limited enrichment, with only one fully significant gene set (Fig. S7E) [47]. Instead, upon separate analyses of the PF or the RF, a strong signature of ibrutinib sensitivity was detected exclusively in the post-ibrutinib RF, whereas the PF at progression turned out virtually identical to the one at pre-treatment (Fig. S7F and Table S3B). This latter observation suggests that ibrutinib inhibition is still efficacious within the RF cells, while it is not within the PF cells, in keeping with an enrichment of resistance-driving mechanisms, either mutation-dependent or mutation-independent. These results highlight the phenotypical similarities between the pre- and post-treatment PF, and demonstrate that the reconstituted PF represents the egressed fraction of a recently proliferated, ibrutinib-resistant CLL cell population.

### *BTK*-unmutated PF at progression is enriched in BCR bypass signaling pathways

We then investigated which pathways could putatively provide ibrutinib resistance in absence of *BTK/PLCG2* mutations. Both *BTK*-mutated and *BTK*-unmutated PF at progression were equally enriched in proliferation-related gene sets indicating low ibrutinib sensitivity (Fig. S8A, B and Table S4A, B). We then evaluated if different transcriptional programs could differentiate the *BTK*-unmutated from *BTK*-mutated PF at progression. Exploratory analyses, using GSEA either through the Hallmark collection, or the C2.cgp collection (C2 curated, chemical and genetic perturbations) (Fig. 4D, Fig. S8C, D and Tables S4C, D), as well as GSVA (Fig. 4E), which enables gene set evaluation at the single-sample level [32], collectively suggested a role of MYC-related signaling and possibly Toll-like receptor 9 (TLR9) signaling in supporting the *BTK*-unmutated PF [50]. Of note, droplet digital PCR and FISH analyses excluded MYC copy number gains as a possible mechanism of pathway activation (Fig. S8E).

On the other hand, the *BTK*-mutated CLL PF showed a relative enrichment of BCR-related gene sets (Fig. 4E), in agreement with the reactivation of the BCR pathway due to the development of *BTK* mutations [51].

To verify whether CLL samples at progression were indeed still responsive to BCR stimulation, we performed in-vitro stimulations with anti-IgM, CD40 ligand (CD40L) or CpG, in presence or not of ibrutinib and measured the phosphorylation of BTK and ERK1/2 proteins by phosphoflow [7]. We also included matched pre-ibrutinib samples for comparison. IgM stimulation was able to induce p-BTK and p-ERK1/2 in all subsets (p-BTK: pre-IBR *p* = 0.02, *BTK*-wt *p* = 0.0035, *BTK*-mut *p* = 0.0028; p-ERK1/2: pre-IBR *p* = 0.0011, *BTK*-wt *p* < 0.001, *BTK*-mut *p* = 0.0026; Fig. S9A), which was counteracted more efficiently by ibrutinib in *BTK*-wt cases (ibrutinib versus untreated, p-BTK: pre-IBR *p* = 0.031, *BTK*-wt *p* = 0.0039, *BTK*-mut *p* = 0.031; p-ERK1/2: pre-IBR *p* = 0.016, *BTK*-wt *p* = 0.0039, *BTK*-mut *p* = 0.031; Fig. S9A). CD40L stimulation was significantly less efficient, eliciting ERK1/2 phosphorylation only in *BTK*-wt cases at progression (*p* = 0.010). Similarly, CpG stimulation was able to induce activation of the MYC pathway particularly in *BTK*-wt cases at progression (Fig. S9B), again suggesting its greater proficiency in these patients.

## Discussion

Several validated biomarkers predicting outcome in ibrutinib-treated CLL patients have been reported so far, usually taken at pre-treatment [7, 39, 41, 42, 52–55]. However, with the exception of *BTK/PLCG2* mutations [3, 4, 56–58], much less information is available regarding dynamic markers whose modulation can inform on disease progression occurring under ibrutinib [11, 44].

In the present study, by taking advantage of two cohorts of ibrutinib-treated CLL patients, we provide evidence that: (i) the CXCR4^dim^/CD5^bright^ PF, suppressed by ibrutinib treatment, reappears upon disease progression, and associates with drug discontinuation due to progression; (ii) the reappearance of the CXCR4^dim^/CD5^bright^ PF occurs independently from *BTK/PLCG2* mutations but, when present, the PF turns out particularly enriched in these lesions; (iii) the transcriptomic profiling of such emerging PF reveals similarities between the pre- and post-treatment fractions, confirming the ibrutinib-resistant and proliferative features of the reconstituted proliferative subpopulation.

During the regular course of CLL, the PF is built up by cells that have just undergone proliferation within the microenvironmental niches within the lymph nodes or the bone marrow [13]. Although the exact stimuli that drive CLL are not yet completely elucidated and may possibly be patient-dependent [14, 19, 59–61], the PF phenotype being a signature of these “just egressed from proliferation sites” cells, has been extensively validated by many studies [14, 15, 17, 46, 48] and also supported by the evidence that inhibition of CLL proliferation by targeted agents such as ibrutinib promptly abolishes this phenotype [8, 9]. However, most of these studies focused on in-vitro and short-term experiments under ibrutinib carried out within the time frame of days or weeks [8–10, 47, 48].

Our study is, to our knowledge, the first report of a long-term quantitative assessment of the modulation of the PF in cohorts of patients treated with ibrutinib monotherapy belonging to either a clinical trial or from real-world diagnostic routine. After the first three years of treatment, with the efficient inhibition of CLL proliferation and the loss of the CXCR4^dim^ CLL population, we were able to describe the reappearance of the CXCR4^dim^/CD5^bright^ PF phenotype especially in patients who were about to discontinue ibrutinib due to progression; in this sense, this rebound advocates for the presence of a novel proliferating subpopulation, allegedly taking place at the level of lymph node/marrow niches, and possibly driven by mechanisms of pharmacological resistance.

In fact, BTK inhibition is rarely maintained indefinitely, and, over time, clinical progression arises [62–66], often associated with acquired resistance mutations of *BTK* and *PLCG2* [3, 4, 56–58]. In our CRO cohort, overall incidence of such mutations was 31%, raising to 58.5% when considering only progressing cases (31/53). Targeted DNA sequencing of the FACS-sorted PF/RF subpopulations revealed an average of 3-fold enrichment of *BTK/PLCG2* mutations in the PF versus the RF, demonstrating that the *BTK*-mutated reservoir is likely resident within the proliferative compartments, where the tumor microenvironment can provide the strongest supportive signals [7, 16, 19, 61]. Of note, two patients (TS33 and RMGEM19) present a few mutations apparently enriched in the RF (two C481R *BTK* mutations), but also carry other mutations strongly enriched in the PF (*BTK* C481Y and C481S), suggesting independent mutational events under clonal selection. Despite the important role of *BTK/PLCG2* mutations, these lesions fail to recapitulate all progression events [24]. In fact, about 40% of cases who ultimately discontinued ibrutinib due to progression, did not show any hotspot mutation on either gene, however presenting a significantly elevated PF.

We then verified whether the PF detected at progression was functionally consistent with an emerging novel proliferative entity. In this regard, here we demonstrate that the same transcriptional signatures that characterize the PF/RF fractions at pre-ibrutinib [13, 15, 17, 46] are equally active in the reconstituted PF at disease progression, and this holds true independently of the *BTK* mutational status. Of note, our transcriptomic data identified as up-regulated in the PF several genes recently associated with CLL proliferation, such as the C-lectin ligand CLECL1 [45], and the B-cell activation marker CD27, recently reported by Takacs et al. [44] as up-regulated at progression in *BTK*-mutated CLL cases. Furthermore, when we tested the extent of ibrutinib sensitivity of the PF/RF fractions at progression, a significant enrichment of genes known to be modulated by ibrutinib [10, 46–49] was present only between the RF fractions, whereas the PF subpopulations appeared virtually insensitive, in agreement with their increase upon progression and the relative enrichment in *BTK/PLCG2* mutations.

The proliferation potential and ibrutinib insensitivity was not exclusive of *BTK*-mutated cases, as also the *BTK*-unmutated samples presented comparable signatures; recent studies reported an increased frequency of mutations in the *BIRC3* and *NFKBIE* genes [24, 67], which may enable a bypass signaling through NF-κB, or possibly through non-BCR-dependent pathways [68]. Indeed, when evaluating if different signaling programs may characterize ibrutinib-resistant *BTK*-unmutated CLL, we could detect a more frequent usage of MYC signaling along with TLR9 signaling. In this regard, *BTK*-unmutated CLL cells, whose BCR signaling is still inhibited by ibrutinib, may try to compensate the decrease of pro-survival signals no longer gained through the BCR pathway by activating other pathways, including MYC and/or TLR9 [69–73]. The presence of a coordinated signals through TLR9 has been reported in other lymphoproliferative diseases [74], where BCR and TLR9 actively cooperate to activate NF-κB. Therefore it can be speculated that CLL cells, after prolonged inhibition of BCR by ibrutinib, may try to adapt to different stimuli that provide the same pro-survival signals [70, 75, 76]. In this context, an unaddressed issue remains the responsiveness of the proliferative compartment to newer-generation BTK inhibitors, which provide better and longer remissions; of note, a single CLL patient (TS208) with high PF at progression, treated with pirtobrutinib for over a year, showed a rapid and stable loss of the PF (FP and AZ, personal observation). Another issue is represented by the gating strategy for the PF, which is not always unambiguous. To account for patient-specific variability, we opted for an individualized gating strategy for the PF, informed on the pre-ibrutinib sample to track the modulation of CXCR4 and CD5 under ibrutinib. In this regard, the use of surrogate markers such as mean fluorescence intensity of CXCR4 or of CD20, to avoid CXCR4/CD5 gating, remains to be established.

In summary, here we report that CLL progression under ibrutinib associates with the reappearance of CXCR4^dim^/CD5^bright^ PF, which precedes clinical progression, increase of serum B2M and ALC counts. Transcriptomic/genetic profiling of the post-ibrutinib PF suggests presence of clonal selection dynamics and active bypass signaling pathways, bona fide taking place within the lymph node microenvironment, which includes *BTK/PLCG2* mutations and the emergence of active bypass signaling pathways. Clinically, as suggested by the flow cytometric longitudinal prospective monitoring in real-world ibrutinib-treated CLL patients presented here, as well as by the fresh/frozen comparisons, the CXCR4^dim^/CD5^bright^ fraction may be viewed as a reproducible and technically robust biomarker to be employed for patient follow-up during ibrutinib therapy. If further validated in external cohorts, it may allow physicians to opt for a more careful monitoring of patients by helping the early detection of CLL progression.

### Supplementary information

Supplemental information

Table S1

Table S2

Table S3

Table S4

## Data Availability

RNA-sequencing data can be accessed to Gene Expression Omnibus at record GSE249956.
