# Supplementary material for: Early reappearance of intraclonal proliferative subpopulations in ibrutinib-resistant chronic lymphocytic leukemia
Source: Leukemia. 2024 Jun 24;38(8):1712–21. doi: 10.1038/s41375-024-02301-y (PMC11286529; doi:10.1038/s41375-024-02301-y)
Supplement: Supplementary file 1 — Supplemental information [file 41375_2024_2301_MOESM1_ESM.pdf]

Supporting Information for

The CXCR4^{dim}/CD5^{bright} Proliferative Fraction Reappears in Ibrutinib-treated Relapsed Chronic Lymphocytic Leukemia Enriched in *BTK/PLCG2* Mutations

- **Supplemental Figures:**
  - Figure S1. CRO cohort characteristics
  - Figure S2. Examples of PF dynamics under ibrutinib in the IOSI cohort.
  - Figure S3. Resting fraction dynamics in the IOSI-EMA-001 trial.
  - Figure S4. PF and white blood cell counts in the CRO cohort.
  - Figure S5. PF dynamics under long-term ibrutinib in the CRO cohort.
  - Figure S6. Cell sorting.
  - Figure S7. Transcriptomic programming of PF/RF subpopulations.
  - Figure S8. Enrichment analysis in BTK-unmutated versus BTK-mutated PF at progression.
  - Figure S9. In-vitro stimulation of CLL cells at progression under ibrutinib.
- **Supplemental Tables:**
  - Table S1. Patients' charateristics of the CRO cohort.
  - Table S2. Gene expression signature of PF and RF at pre-treatment.
  - Table S3. Gene Set Enrichment Analysis of proliferation and ibrutinib gene sets.
  - Table S4. Gene Set Enrichment Analysis of PF at progression.

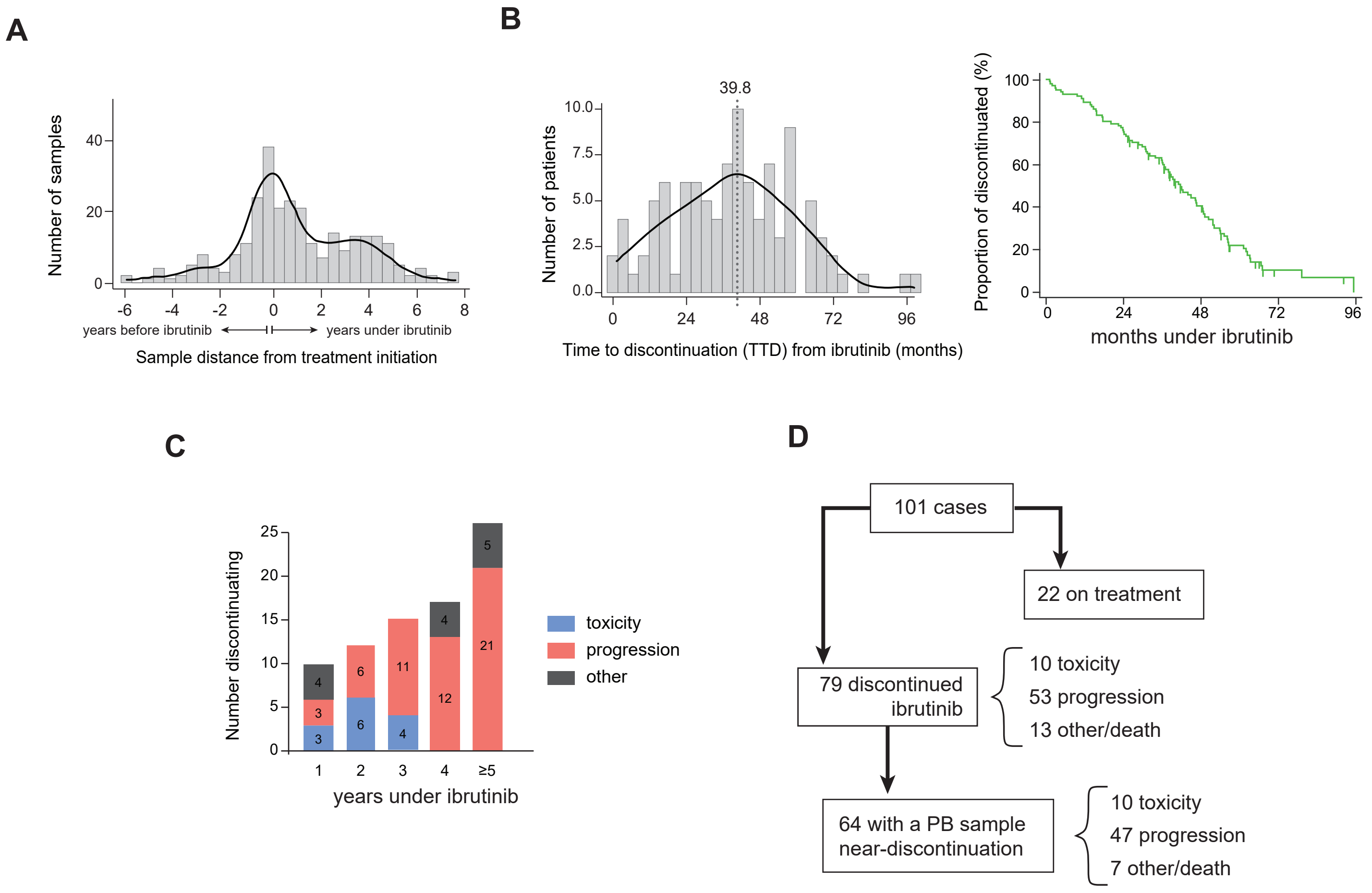

Figure S1. CRO cohort characteristics. (A) Histogram of distribution of 300 samples according to the time from ibrutinib initiation. **(B)** Left Panel: Histogram of distribution of time under ibrutinib of all 101 cases; median time to discontinuation is 39.8 months. Right panel: Kaplan-Meyer curve of time to ibrutinib discontinuation. **(C)** Stacked bar plot of frequency of ibrutinib discontinuations, stratified by year under therapy and by cause of discontinuation. **(D)** Consort diagram of ibrutinib discontinuation events.

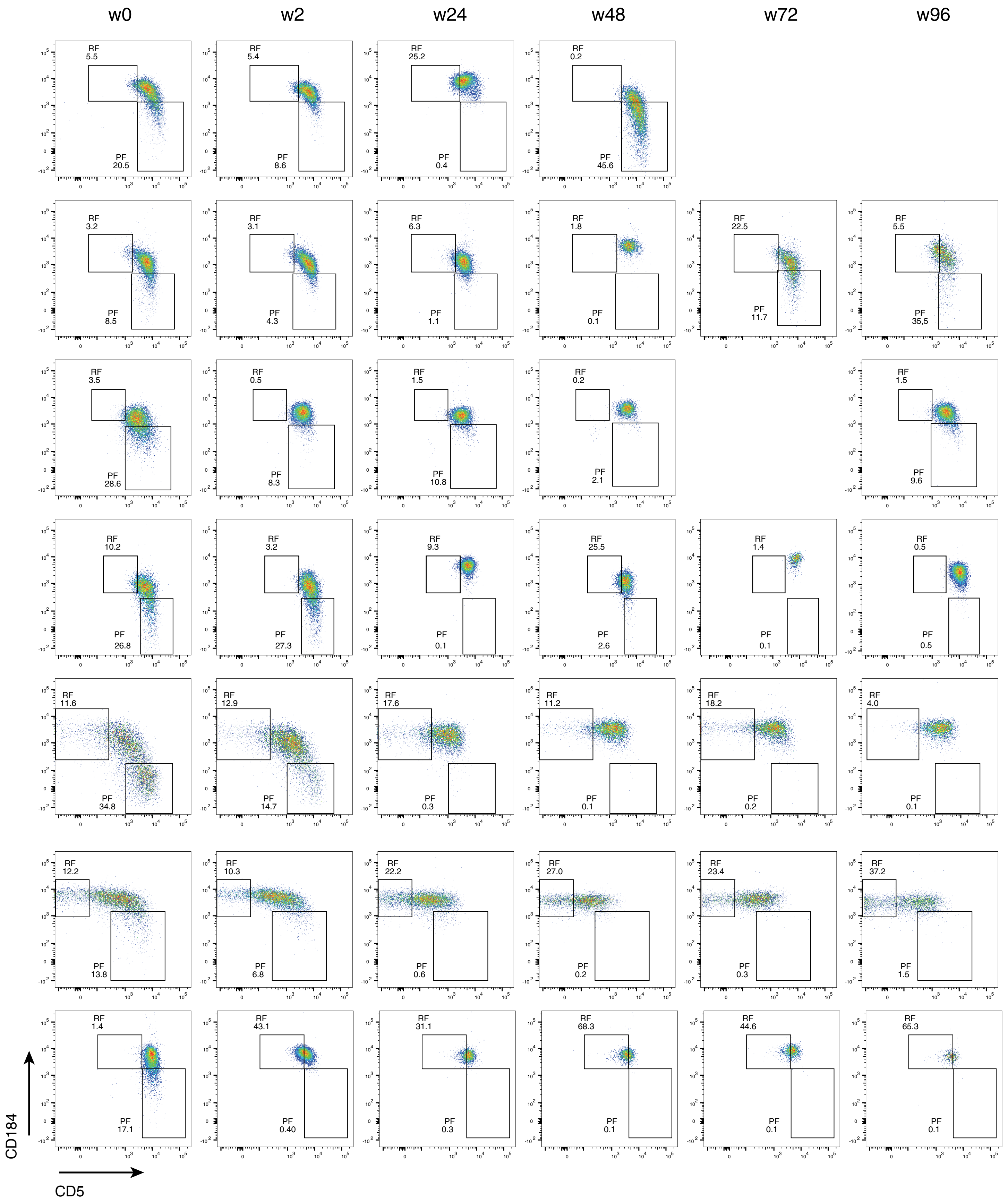

Figure S2. Examples of PF dynamics under ibrutinib in the IOSI cohort.

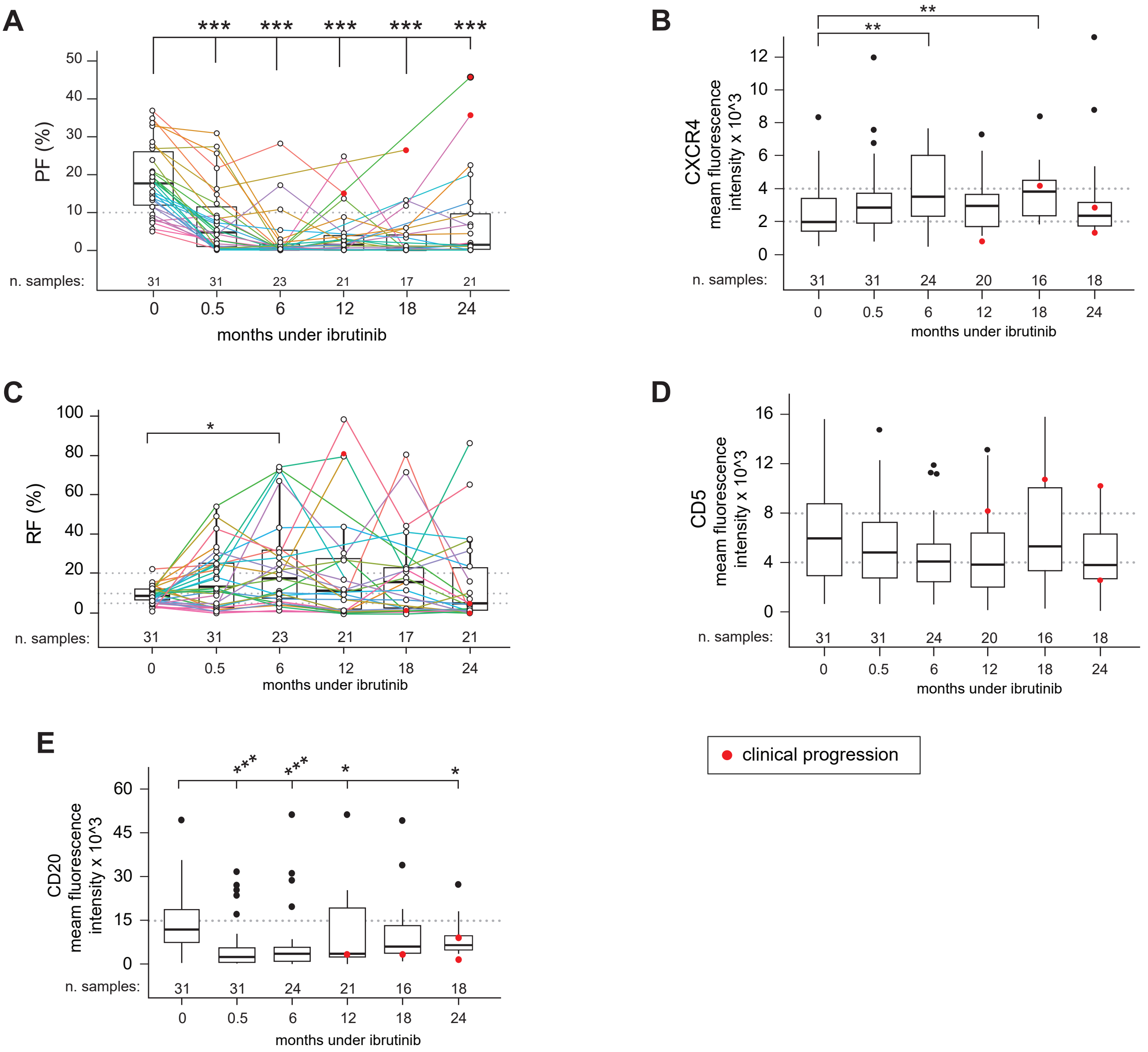

Figure S3. PF-RF fraction dynamics in the IOSI-EMA-001 trial. Box-and-whiskers plot of the proliferating fraction PF **(A)**, CXCR4 expression **(B),** resting fraction RF **(C)**, CD5 expression **(D)** and CD20 expression **(E)** over time by month under ibrutinib. *** p≤0.001, ** p≤0.01, * p≤0.05 by two-sided Mann-Whitney rank-test, compared to month zero. Only significant comparisons are reported. Lines connect samples belonging to the same patient. Red dots indicate samples collected from patients in clinical progression.

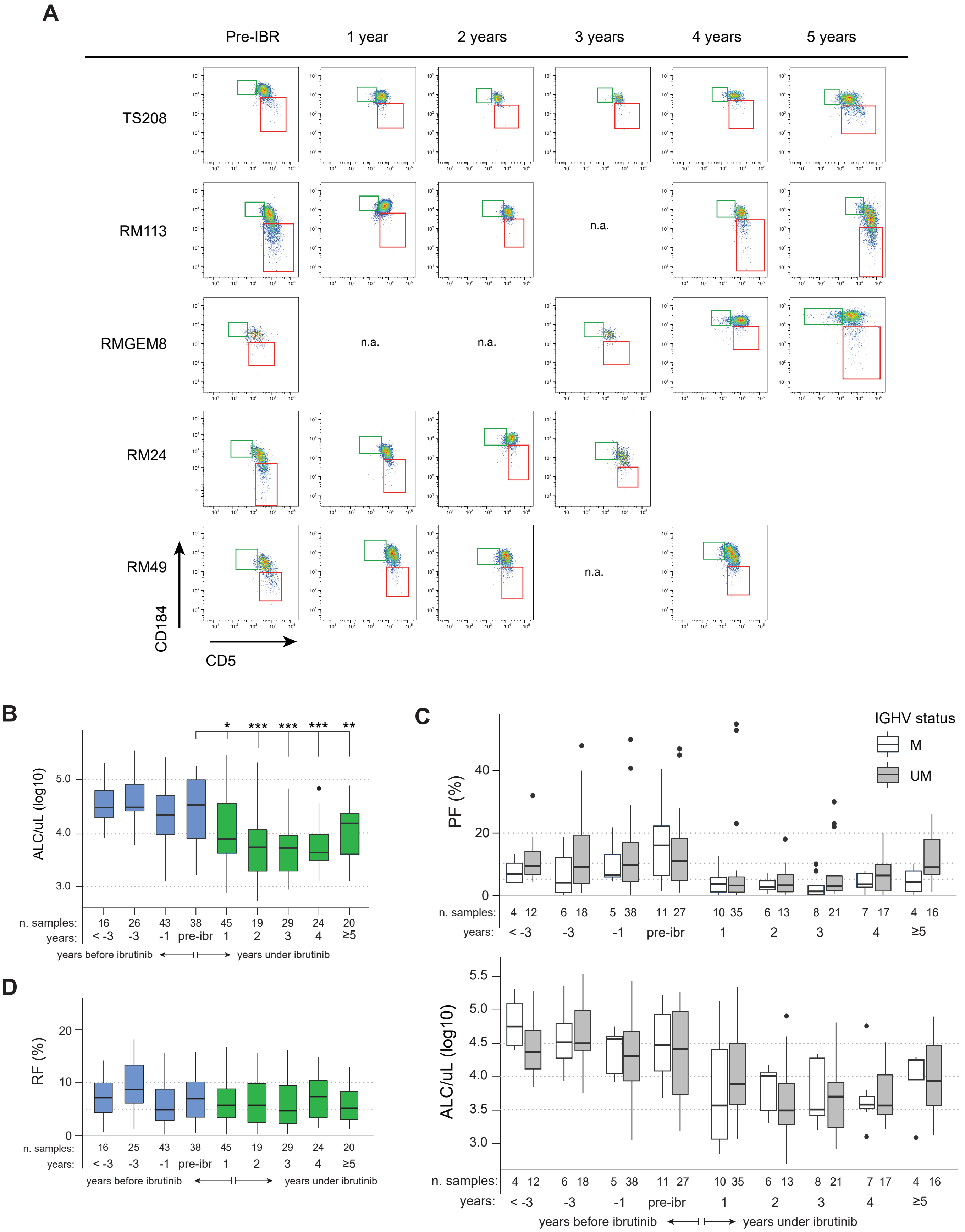

Figure S4. PF and lymphocytes counts in the CRO cohort. (A) Prospective analysis of selected cases from the CRO cohort reporting the gating strategy for PF and RF in samples collected at the indicated year under ibrutinib therapy. n.a.: sample not available. **(B)** Box-and-whiskers plot of the absolute lymphocytes count (ALC) in samples from the CRO cohort before (blue) and after (green) ibrutinib initiation. Samples are grouped by number of years under ibrutinib; pre-ibrutinib samples have been collected within 6 months prior to therapy initiation. **(C)** Box-and-whiskers plot of PF and ALC in the CRO cohort, stratified by IGHV status. Statistical significance was computed at each time point with Wilcoxon paired rank test of IGHV-M versus IGHV-UM. No significant comparison was found. **(D)** Box-and-whiskers plot of the resting fraction (RF) in the CRO cohort before (blue) and after (green) ibrutinib initiation. No significant comparison was found. Dotted lines are added for visual reference.

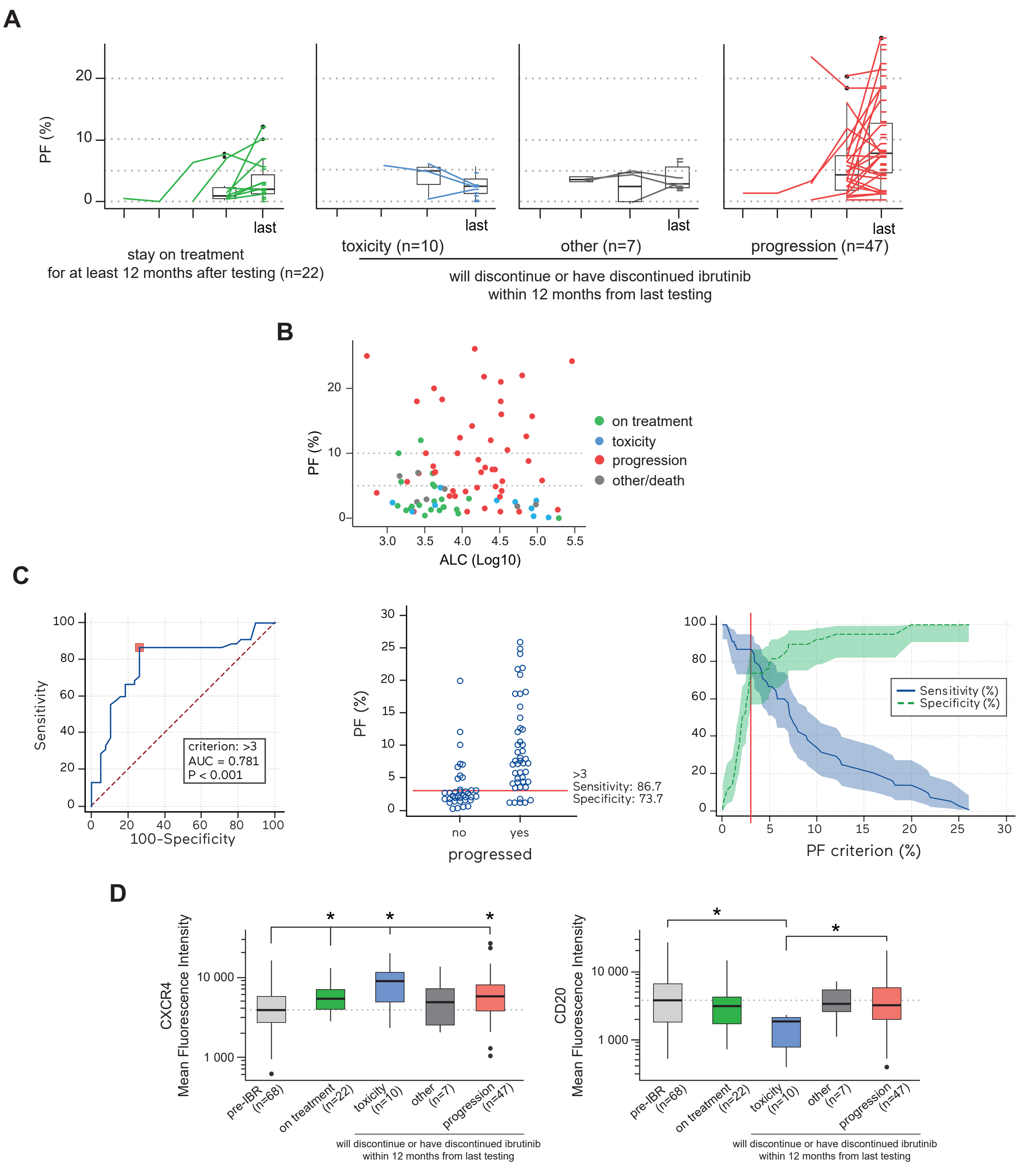

Figure S5. PF dynamics under long-term ibrutinib in the CRO cohort. (A) Dot-and-line plot of the PF in 22 on-treatment cases and 64 cases with a near-discontinuation samples ("near-discontinuation cohort"), stratified by cause of ibrutinib interruption. Each line represents a single CLL case, plotting all available samples collected under ibrutinib, right-aligned to the last sample available (number of segments therefore reflects the number of samples for each case). **(B)** Dot plot of the PF versus absolute lymphocytes count (ALC) in near-discontinuation and on-treatment samples. **(C)** Left panel: Receiver Operating Charateristic (ROC) curve for PF to predict clinical progression (progression versus all other categories) in the near-discontinuation cohort (n=86); Middle panel: dot plot of PF in progressed or non-progressed cases. Right panel: sensitivity and specificity of clinical progression according to increasing PF criterion. Red line denotes the criterion (PF>3%) selected by ROC analysis. **(D)** Distribution of mean fluorescence intensity of CXCR4 (left panel) or CD20 (right panel) in the near-discontinuation samples, split by cause of discontinuation, compared to the on-treatment samples and with matched pre-ibrutinib samples, if available (n=68). Dotted lines are added for visual reference. * p≤ 0.05, by two-sided Mann-Whitney rank-test. Only significant comparisons are reported.

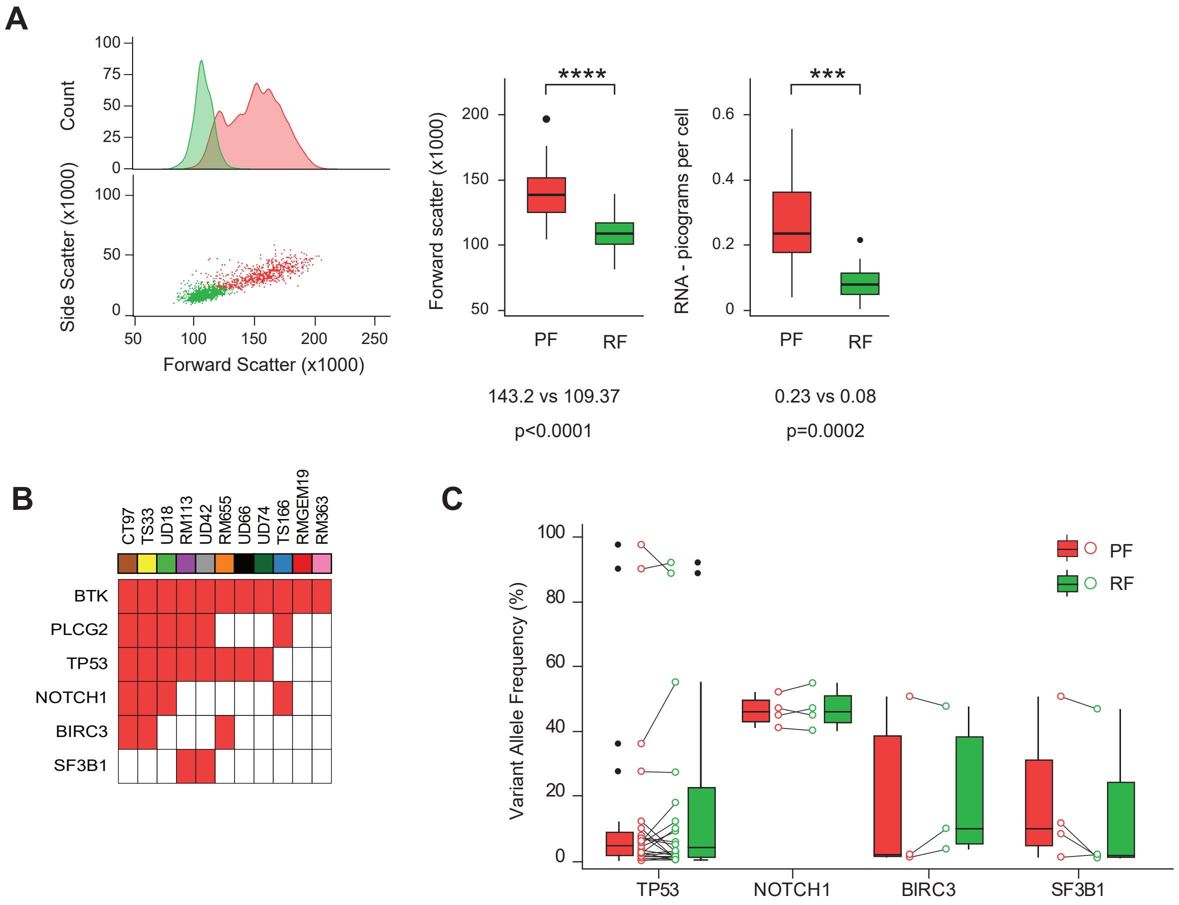

Figure S6. Cell sorting. (A) Left panel: forward scatter intensity histogram (upper) and dot plot versus side scatter (lower) of PF (red) and RF (green) from a representative sorted sample. Central panel: box-and-whiskers plot of forward scatter intensity in all sorted PF and RF subpopulations. Right panel: box-and-whiskers plot of RNA yield from all sorted PF and RF subpopulations. **(B)** Oncoplot of all sorted samples with detected somatic mutations in recurrently mutated genes. **(C)** Box-and-whiskers and dot-and-line plots of variant allele frequency of somatic mutations detected in the sorted PF and RF subpopulations. Comparison of PF versus RF was performed with Wilcoxon paired rank test of PF versus RF; comparisons were all not significant.

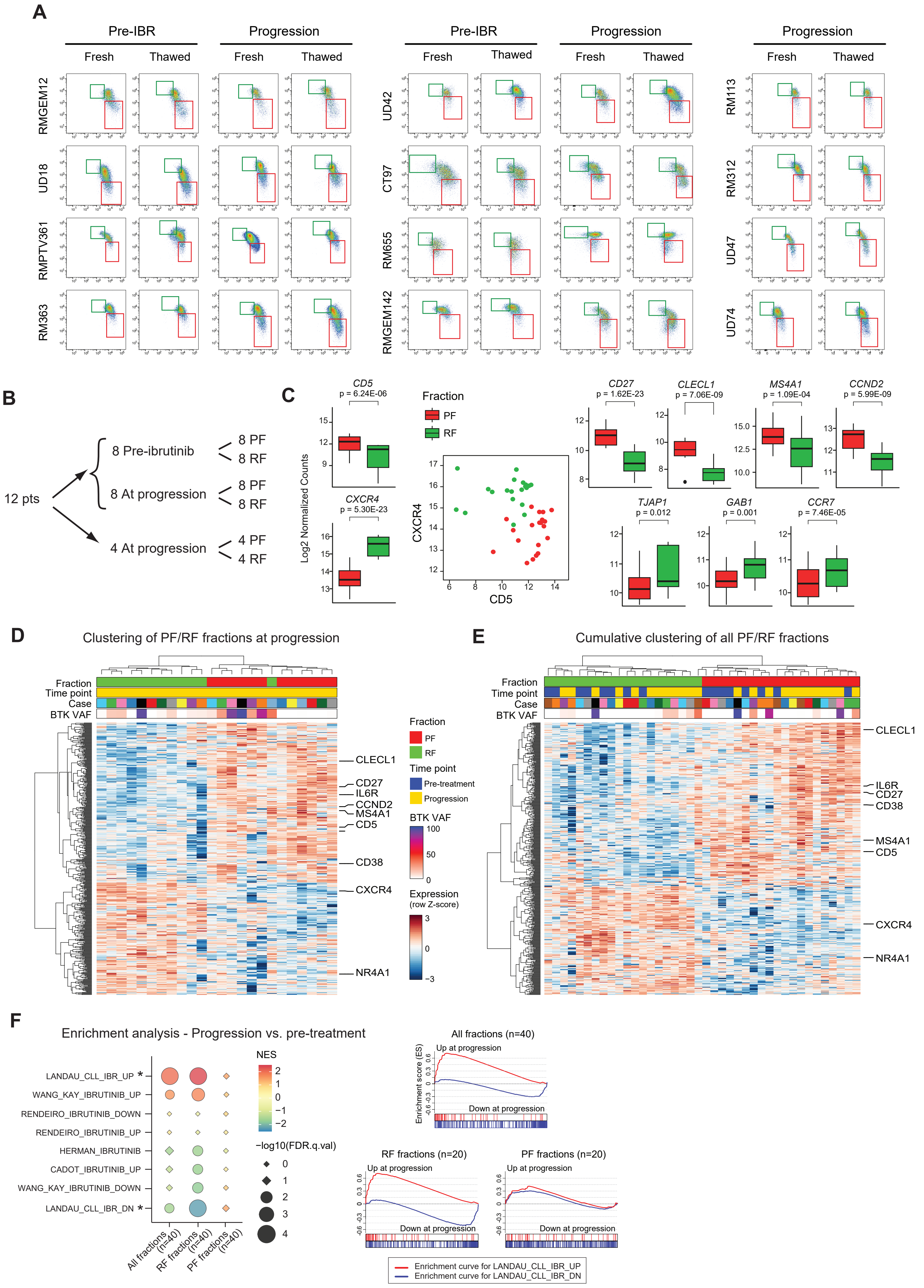
A
Pre-IBR
Progression
Fresh
Thawed
RMGEM12
UD18
RMPTV361
RM363
UD42
CT97
RM655
RMGEM142
RM113
RM312
UD47
UD74
B
12 pts
8 Pre-ibrutinib
8 At progression
4 At progression
8 PF
8 RF
4 PF
4 RF
C
Log2 Normalized Counts
CD5
p = 6.24E-06
CXCR4
p = 5.30E-23
Fraction
PF
RF
CXCR4
CD5
CD27
p = 1.62E-23
CLECL1
p = 7.06E-09
MS4A1
p = 1.09E-04
CCND2
p = 5.99E-09
TJAP1
p = 0.012
GAB1
p = 0.001
CCR7
p = 7.46E-05
D
Clustering of PF/RF fractions at progression
Fraction
Time point
Case
BTK VAF
CLECL1
CD27
IL6R
CCND2
MS4A1
CD5
CD38
CXCR4
NR4A1
Fraction
PF
RF
Time point
Pre-treatment
Progression
BTK VAF
Expression
(row Z-score)
E
Cumulative clustering of all PF/RF fractions
CLECL1
IL6R
CD27
CD38
MS4A1
CD5
CXCR4
NR4A1
F
Enrichment analysis - Progression vs. pre-treatment
LANDAU_CLL_IBR_UP *
WANG_KAY_IBRUTINIB_UP
RENDEIRO_IBRUTINIB_DOWN
RENDEIRO_IBRUTINIB_UP
HERMAN_IBRUTINIB
CADOT_IBRUTINIB_UP
WANG_KAY_IBRUTINIB_DOWN
LANDAU_CLL_IBR_DN *
All fractions (n=40)
RF fractions (n=40)
PF fractions (n=40)
NES
−log10(FDR.q.val)
All fractions (n=40)
Enrichment score (ES)
Up at progression
Down at progression
RF fractions (n=20)
PF fractions (n=20)
Enrichment curve for LANDAU_CLL_IBR_UP
Enrichment curve for LANDAU_CLL_IBR_DN

Figure S7. Transcriptomic programming of PF/RF subpopulations. (A) Comparison of PF/RF phenotype in samples used for RNA-seq experiments performed on fresh blood at sample's arrival (Fresh) compared to the one from cell sorting (Thawed). **(B)** Disposition of CLL samples sorted for RNA-seq. **(C)** Box-and- whiskers plots of log2 normalized counts of representative genes from the 476-genes signature of PF versus RF at pre-ibrutinib. Reported are adjusted p-values from differential expression analysis with Deseq2. Inset: scatter plot of CD5 and CXCR4 transcript counts from all (n=40) sorted fractions, recapitulating the CD5/CXCR4 phenotype by flow cytometry. **(D)** Unsupervised clustered heatmap of the PF/RF 476-genes signature, evaluated in the PF/RF subpopulations at progression (n=24). **(E)** Unsupervised clustered heatmap of the PF/RF 476-genes signature, evaluated in all (n=40) PF/RF subpopulations at pre-ibrutinib (n=16) and at progression (n=24). Annotation labels indicate PF/RF subpopulations, type of sample, case and *BTK* VAF, if mutated. **(F)** Summary dot-plot of Gene Set Enrichment Analysis (GSEA) on custom gene sets of ibrutinib signature curated from literature (Herman et al. ref.6, Landau et al. ref.47, Cadot et al. ref.46, Wang et al. ref.48). Color represents the normalized enrichment score, size is proportional to –log10(FDR q-value), shape denotes significance (circle if q-value<0.05). Enrichment plots of marked (*) gene sets are reported for all fractions or for the PF/RF subpopulations, separately analyzed. GSEA summary is reported in Table S3B.

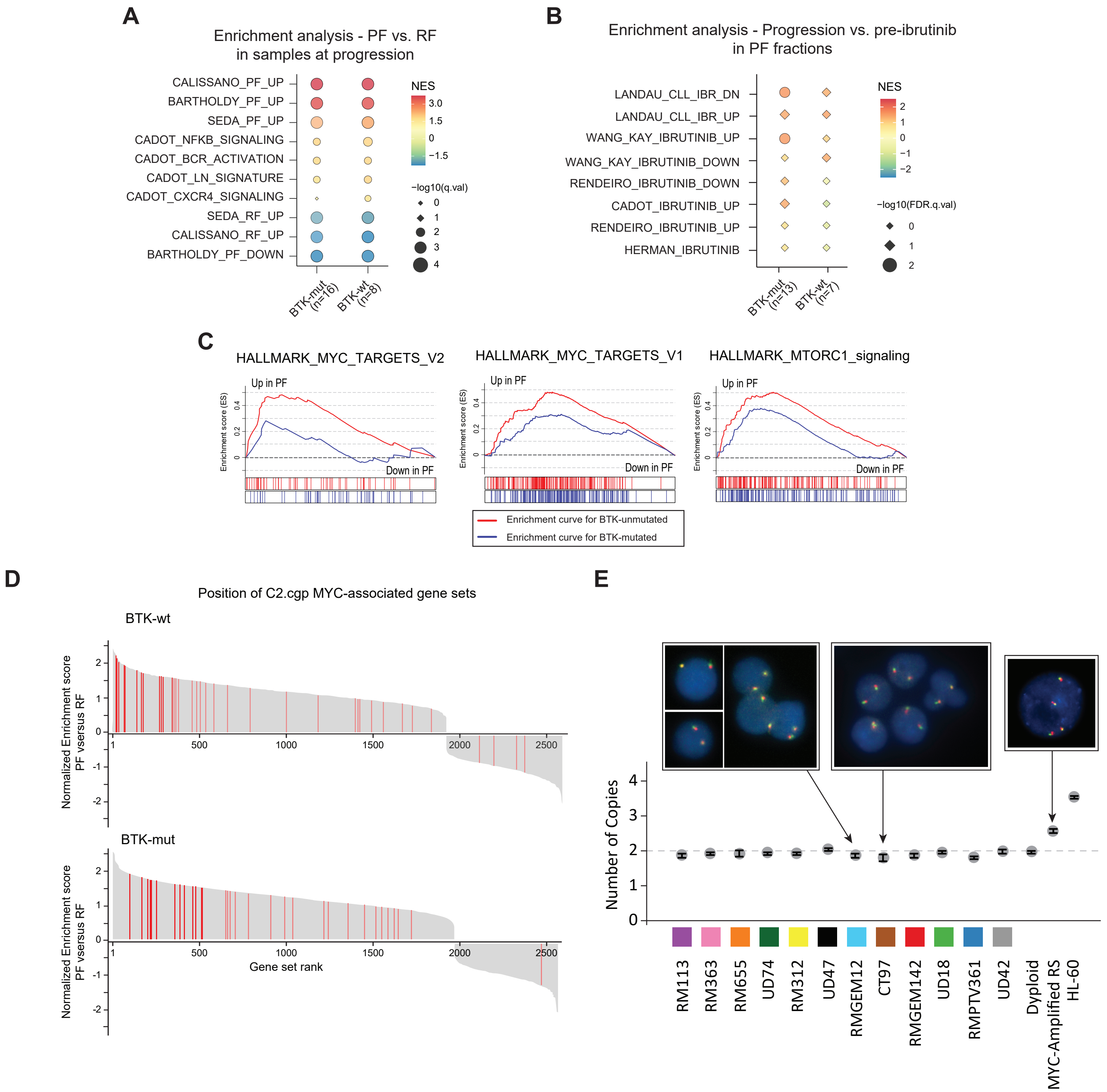

Figure S8. Enrichment analysis in *BTK*-unmutated versus *BTK*-mutated PF at progression. (A) Summary dot-plot of Gene Set Enrichment Analysis (GSEA) on PF/RF-related gene sets curated from literature (Calissano et al. ref.13, Bartholdy et al., ref.15, Cadot et al. ref.46, Seda et al. ref.17). Color represents the normalized enrichment score, size is proportional to –log10(FDR q-value), shape denotes significance (circle if q-value<0.05). GSEA summary is reported in Table S4A. **(B)** Summary dot-plot of Gene Set Enrichment Analysis (GSEA) on custom gene sets of ibrutinib signature curated from literature (Herman et al. ref.6, Landau et al. ref.47, Cadot et al. ref.46, Wang et al. ref.48). Color represents the normalized enrichment score, size is proportional to –log10(FDR q-value), shape denotes significance (circle if q-value<0.05). GSEA summary is reported in Table S4B. **(C)** Enrichment plot from GSEA of the HALLMARK_MYC_TARGETS_V2 and HALLMARC_MTORC1_SIGNALING of PF versus RF in *BTK*-unmutated (red curves) versus *BTK*-mutated PF (blue curves) at progression. GSEA summary for whole Hallmark collection is reported in Table S4D. **(D)** Waterfall plot of Normalized Enrichment Scores from PF versus RF GSEA on the C2.cgp gene sets collection, within *BTK*-unmutated (upper panel) or within *BTK*-mutated (lower panel) cases at progression. Red lines represent scores of MYC-related gene sets. GSEA summary with highlighted gene sets is reported in Table S4E.
(E) *MYC* copy number analysis with droplet digital PCR and FISH break-apart probe. Dyploid: reference normal DNA; RS: Richter Syndrome.

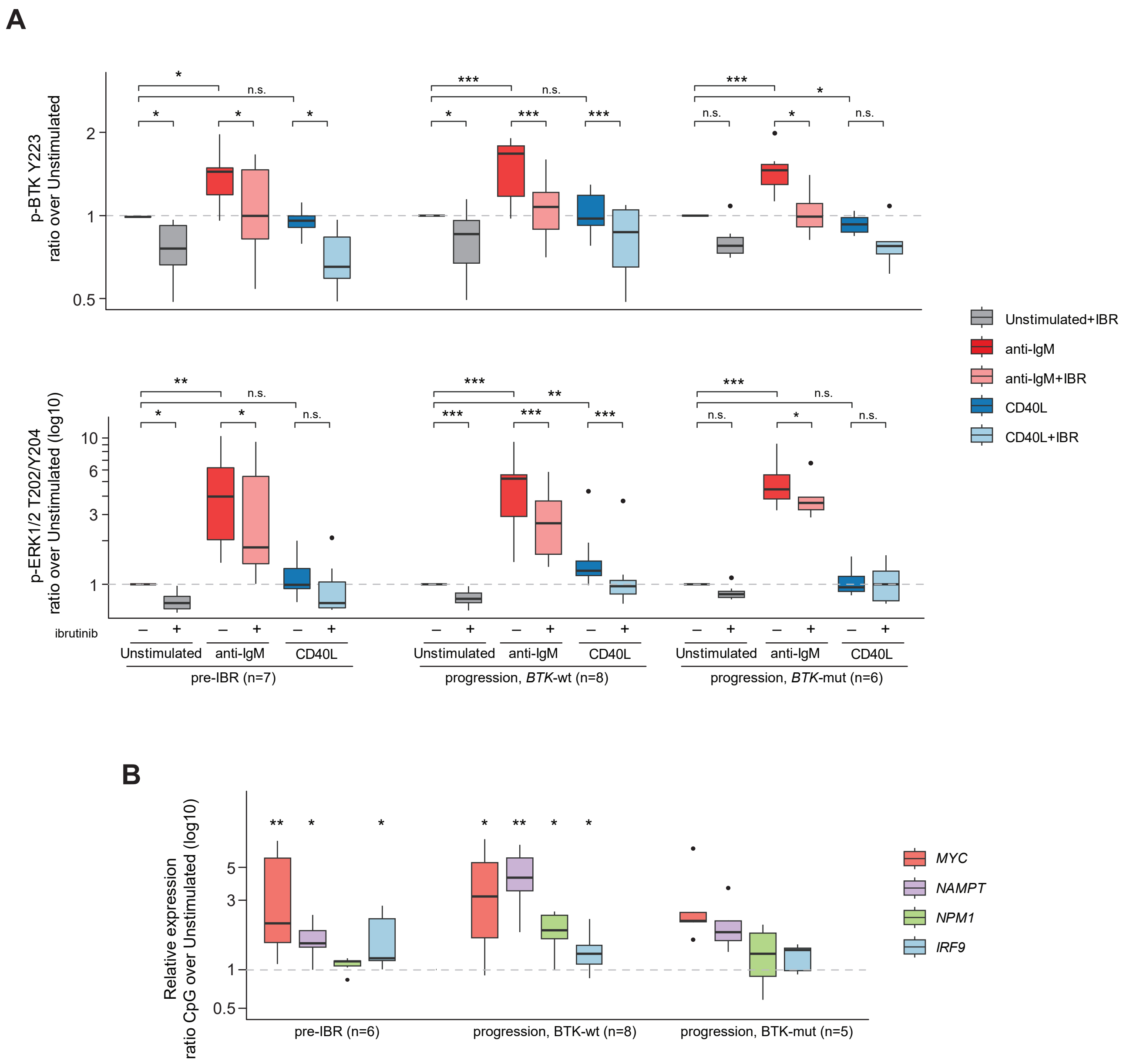

Figure S9. In-vitro stimulation of CLL cells at progression under ibrutinib. (A) Box and whiskers plot of phospho-BTK (Tyr223; upper panel) or phospho-ERK1/2 (Thr202/Tyr204; lower panel) determined by flow cytometry, in CLL stimulated with anti-IgM conjugated beads or CD40 ligand in presence or not of ibrutinib. Data are presented as ratio of the stimulated condition versus unstimulated. Matched pre-ibrutinib samples were included for comparison if cryopreserved cells were available. **(B)** Box and whiskers plot of relative expression of *MYC* and MYC-target genes, determined by real-time PCR, in CLL stimulated with CpG+IL2. Data are presented as ratio of the stimulated condition versus unstimulated.

Matched pre-ibrutinib samples were included for comparison if cryopreserved cells were available. n.s. not significant, * p<0.05, ** p<0.01, *** p<0.005 by Wilcoxon paired test.
